# Medical therapy and outcomes in REVIVED-BCIS2 and STICHES: an individual patient data analysis

**DOI:** 10.1093/eurheartj/ehaf080

**Published:** 2025-03-06

**Authors:** Matthew Ryan, Mark C Petrie, Evangelos Kontopantelis, Matthew Dodd, Guangyu Tong, Guillaume Marquis-Gravel, Kieran F Docherty, Tim Clayton, Alexandra J Lansky, Mamas A Mamas, Jean-Lucien Rouleau, Eric J Velazquez, Divaka Perera

**Affiliations:** Kings College London, British Heart Foundation Centre of Research Excellence, 4th Floor Lambeth Wing, St Thomas’ Hospital, Westminster Bridge Road, London SE1 7EH, UK; Cardiovascular Division, Guy's and St Thomas' NHS Foundation Trust, London, UK; School of Cardiovascular and Metabolic Health, University of Glasgow, Glasgow, Scotland, UK; Division of Informatics, Imaging and Data Sciences, The University of Manchester, Manchester, UK; Department of Medical Statistics, London School of Hygiene & Tropical Medicine, London, UK; Cardiovascular Medicine Analytics Center, Yale School of Medicine, New Haven, CT, USA; Department of Medicine, Montreal Heart Institute, Université de Montréal, Montreal, QC, Canada; School of Cardiovascular and Metabolic Health, University of Glasgow, Glasgow, Scotland, UK; Department of Medical Statistics, London School of Hygiene & Tropical Medicine, London, UK; Cardiovascular Medicine Analytics Center, Yale School of Medicine, New Haven, CT, USA; Department of Internal Medicine, Yale School of Medicine, New Haven, CT, USA; Keele Cardiac Research Group, Keele University, Stoke-on-Trent, UK; National Institute for Health and Care Research (NIHR) Birmingham Biomedical Research Centre, Birmingham, UK; Department of Medicine, Montreal Heart Institute, Université de Montréal, Montreal, QC, Canada; Department of Internal Medicine, Yale School of Medicine, New Haven, CT, USA; Kings College London, British Heart Foundation Centre of Research Excellence, 4th Floor Lambeth Wing, St Thomas’ Hospital, Westminster Bridge Road, London SE1 7EH, UK; Cardiovascular Division, Guy's and St Thomas' NHS Foundation Trust, London, UK

**Keywords:** Heart failure, Ischaemic heart disease, Coronary artery disease, Revascularization

## Abstract

**Background and Aims:**

In the Surgical Treatment for Ischaemic Heart Failure Trial Extension Study (STICHES), coronary artery bypass grafting (CABG) improved outcomes of patients with ischaemic left ventricular dysfunction receiving medical therapy, whereas in the Revascularization for Ischaemia Ventricular Dysfunction trial (REVIVED-BCIS2), percutaneous coronary intervention (PCI) did not. The aim of this study was to explore differences in outcomes of participants treated with medical therapy alone in STICHES vs. REVIVED-BCIS2 and to assess the incremental benefit of CABG or PCI.

**Methods:**

Pooled analysis of adjusted individual participant data from two multicentre randomized trials. All patients had left ventricular ejection fraction ≤35% and coronary artery disease and received medical therapy. Participants were randomized 1:1 to CABG (STICHES) or PCI (REVIVED-BCIS2). The primary outcome was the composite of all-cause death and hospitalization for heart failure over all available follow-up.

**Results:**

A total of 1912 participants (88% male, 76% white ethnicity) were included with 98.3% completeness of follow-up for the primary outcome. The median follow-up was 118 months in STICHES and 41 months in REVIVED-BCIS2. Those receiving medical therapy alone in REVIVED-BCIS2 had fewer primary outcome events than those receiving medical therapy alone in STICHES (adjusted hazard ratio 0.60, 95% confidence interval 0.48–0.74, *P* < .001). Patients receiving PCI in REVIVED-BCIS2 were less likely to experience a primary outcome event than those receiving CABG in STICHES. Adjusted outcomes of patients treated with CABG in STICHES were worse than those receiving medical therapy alone in REVIVED-BCIS2.

**Conclusions:**

Patients with ischaemic cardiomyopathy receiving medical therapy in REVIVED-BCIS2 had better outcomes than those in STICHES, with or without CABG surgery. Further trials comparing CABG, PCI, and medical therapy in this population are warranted.

## Structured Graphical Abstract

**Structured Graphical Abstract:**
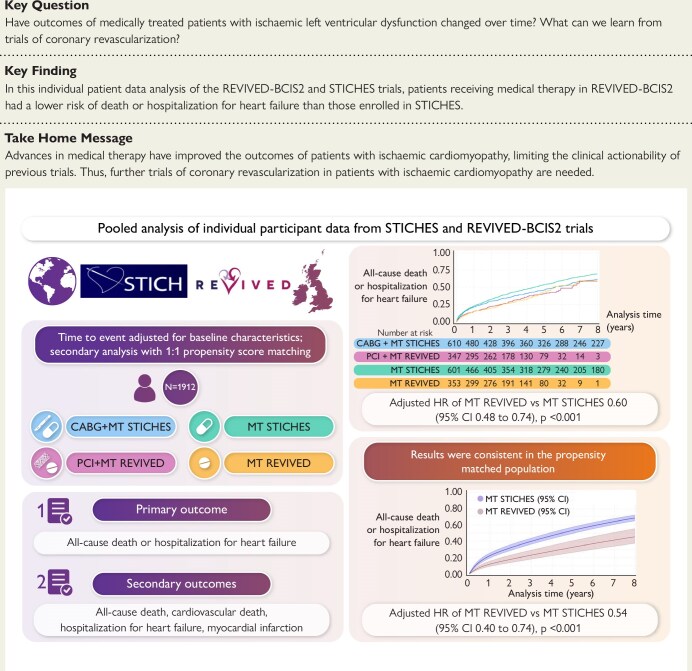
In this pooled individual participant data analysis of 1912 patients with ischaemic left ventricular dysfunction enrolled in REVIVED-BCIS2 and STICHES, patients receiving medical therapy in REVIVED-BCIS2 had better outcomes than those enrolled in STICHES, regardless of treatment assignment. CABG, coronary artery bypass grafting; CI, confidence interval; HR, hazard ratio; MT, medical therapy; PCI, percutaneous coronary intervention; REVIVED-BCIS2, Revascularization for Ischaemia Ventricular Dysfunction Trial; STICHES, Surgical Treatment for Ischaemic Heart Failure Trial Extension Study.

**See the editorial comment for this article ‘Mode of revascularization for ischaemic cardiomyopathy: comparing apples with oranges?’, by K. Chiew *et al*., https://doi.org/10.1093/eurheartj/ehaf201.**

## Introduction

Ischaemic left ventricular (LV) dysfunction is a common cause of heart failure and is increasing in incidence.^1^ There have been major prognostic advances in medical and device therapy in recent decades, but morbidity and mortality rates remain high.^2,3^ Removing the substrate for ischaemia by treating diseased coronary arteries has been hypothesized to improve outcomes in this population, but the only two randomized trials to have addressed this yielded discordant results.^4–7^ In extended follow-up of the Surgical Treatment for Ischaemic Heart Failure trial (STICHES), recruiting participants between 2002 and 2007, treatment with coronary artery bypass grafting (CABG) reduced all-cause mortality over 10-year follow-up compared with medical therapy, though no difference was observed at the initial 5-year analysis.^5,6,8^ In contrast, in the Revascularization for Ischaemic Ventricular Dysfunction trial (REVIVED-BCIS2), recruiting from 2013 to 2020, treatment with percutaneous coronary intervention (PCI) did not reduce the combined primary endpoint of all-cause mortality and hospitalization for heart failure at a median of 3.4 years from randomization.^7,9^ It is unknown whether the contrasting outcomes of REVIVED-BCIS2 and STICHES were due to the different modes of revascularization, advances in medical therapy or differences in patient characteristics, and whether CABG would still provide incremental benefit over the contemporary medical therapy used in REVIVED-BCIS2. The purpose of this study was to use a harmonized dataset combining full individual participant data from STICHES and REVIVED-BCIS2 to (i) compare the clinical outcomes of patients treated with medical therapy alone in STICHES and REVIVED-BCIS2, (ii) estimate whether CABG would provide incremental benefit over more contemporary medical therapy used in REVIVED-BCIS2, and (iii) compare outcomes between CABG and PCI in patients with ischaemic LV systolic dysfunction.

## Methods

### Trial populations and randomized treatments

This pooled analysis combined individual participant data from the STICHES and REVIVED-BCIS2 trials. The rationale, design, and primary results of STICHES and REVIVED-BCIS2 have been published previously; both were prospective, multicentre, open-label, randomized controlled trials.^5–9^ Patients were eligible for enrolment in STICHES if they had LV ejection fraction (EF) ≤35% and any coronary artery disease (CAD) suitable for revascularization, and for REVIVED-BCIS2 if they had LVEF ≤35%, extensive CAD (British Cardiovascular Intervention Society Jeopardy Score ≥ 6), and evidence of viability in at least four dysfunctional myocardial segments which were amenable to treatment with PCI.^10^ Full details of inclusion and exclusion criteria for each trial are included in Supplementary data online, *Table S1*. Randomization was on a 1:1 basis using permuted blocks.

All patients in both trials received medical and device therapy based on guidelines from trial medical therapy committees who updated recommendations on an ongoing basis. In STICHES, patients randomized to revascularization underwent treatment with CABG, including at least one internal mammary artery graft where possible. In REVIVED-BCIS2, patients randomized to revascularization underwent PCI of all significant coronary lesions in major proximal vessels supplying viable myocardium.

### Outcomes and follow-up

All patients were followed up in person at regular intervals for clinical and imaging outcomes. Baseline characteristics, details of assigned treatment, and follow-up data were recorded using dedicated case report forms (CRFs). Outcomes collected included all-cause death, cardiovascular death, hospitalization for heart failure, myocardial infarction, and LVEF. In both trials, primary and key secondary outcome events were adjudicated by independent clinical events committees. Outcome definitions for each trial are included in Supplementary data online, *Table S2*. In STICHES, extended follow-up continued for a minimum of 3.5 years and a maximum of 13.4 years; data from the full duration of follow-up were included in this analysis. In REVIVED-BCIS2, follow-up continued for a minimum of 2.0 years and a maximum of 8.7 years.

The primary outcome of this pooled analysis was the composite of all-cause death or hospitalization for heart failure. Secondary outcomes were individual components of the primary outcome, cardiovascular death, and myocardial infarction.

### Combining individual participant data

Data from the two trial CRFs were combined into a harmonized dataset. Full 10-year data from the STICHES trial were used in preference to the initial 5-year follow-up, in order to utilize the maximum amount of data, recognizing that the follow-up duration would differ between trials regardless of the source of data used. The two trial CRFs were reviewed to identify common variables (baseline and outcomes), and a harmonized dataset was assembled. Outcome definitions in each trial were reviewed to ensure consistency (see Supplementary data online, *Table S2*). Participants were divided into four groups based on trial enrolment and treatment assignment: CABG + medical therapy in STICHES (CABG + MT STICHES), medical therapy alone in STICHES (MT STICHES), PCI + medical therapy in REVIVED-BCIS2 (PCI + MT REVIVED), and medical therapy alone in REVIVED-BCIS2 (MT REVIVED). Missing baseline data were imputed after the datasets were combined using multiple imputation by chained equations, assuming missingness was at random and stratified by trial. These imputed data were included in the reported baseline characteristics, adjustment, and propensity matching.

### Statistical analysis

In accordance with our hypotheses, between-group comparisons of interest were as follows: (i) MT REVIVED vs. MT STICHES, (ii) PCI + MT REVIVED vs. CABG + MT STICHES, and (iii) MT REVIVED vs. CABG + MT STICHES and (iii) PCI + MT REVIVED vs. CABG + MT STICHES. Between-trial differences in baseline characteristics were compared with unpaired *t*-tests or Mann–Whitney tests, depending on the normality of distribution. Kaplan–Meier survival plots were used to estimate and present cumulative event rates for all four groups. Parametric survival models using the Weibull method were used to examine differences in the outcome across the four groups of interest. We used a maximum likelihood estimation parametric regression survival time model, with a Weibull distributional form of the error term. We report hazard ratios (HRs) and 95% confidence intervals (CIs) with MT STICHES as the reference group and all four groups included in the model. The proportional hazards assumption was evaluated using log–log plots.

Outcomes were analysed as intention-to-treat on a time-to-first event basis, with time measured from randomization to the first event or censoring. All analyses were adjusted for established determinants of risk, including age, sex, ethnicity, body mass index, systolic blood pressure, diastolic blood pressure, heart rate, current smoking, hypertension, diabetes, history of myocardial infarction, previous PCI, previous CABG, chronic kidney disease, peripheral vascular disease, presence of an implantable cardioverter defibrillator, LVEF, LV end-systolic volume index, number of diseased coronary arteries, New York Heart Association functional class, Canadian Cardiovascular Society angina grade, treatment with angiotensin-converting enzyme inhibitor, and treatment with beta-blocker.

A sensitivity analysis was carried out using nearest neighbour propensity score matching for each pairwise comparison of interest. The propensity score was calculated only on age, sex, diabetes, chronic kidney disease, extent of CAD, and LVEF, to ensure good balance on the covariates that were a priori considered to be the most clinically important. The approach implemented 1-to-*N* nearest neighbour matching with no replacement, allowing multiple matches from the larger of the two groups within a 0.05 calliper width. Following the matching processes, we used parametric survival models (one for each pairwise comparison) controlling for all covariates previously mentioned (including those used for the generation of the propensity score). As REVIVED-BCIS2 participants were recruited in the UK, we performed a sensitivity analysis with a subset of STICHES patients recruited in comparable healthcare systems (North America, Western Europe, and Poland). All analyses were performed with Stata, version 18.

## Results

A total of 1912 participants were included in the pooled dataset, 610 CABG + MT STICHES, 602 MT STICHES, 347 PCI + MT REVIVED, and 353 MT REVIVED. Enrolment to STICHES was from 24 July 2002 to 5 May 2007 and to REVIVED-BCIS2 from 28 August 2013 to 19 March 2020. The median age was 63.6 [standard deviation (SD) 10.2] years, 1678 (87.8%) participants were male, and 451 (23.6%) were of non-white ethnicities. The mean LVEF was 26.7% (SD 6.3%). Details of the missing data are included in Supplementary data online, *Table S3*. In REVIVED-BCIS2, 12 (3.7%) patients assigned to PCI did not undergo the procedure, whilst 37 (10.5%) of patients in the MT arm underwent unplanned revascularization. In STICHES, 55 patients (9%) assigned to CABG did not undergo the procedure, whilst 119 (19.8%) had CABG performed at any point before the completion of long-term follow-up, 66 (11%) of whom were within 1 year of enrolment.

Compared with STICHES, patients enrolled in REVIVED-BCIS2 were older, equally likely to be male, and less likely to be of non-white ethnicity, had a higher prevalence of chronic kidney disease and a lower prevalence of prior myocardial infarction (*Table 1*). Enrolment to REVIVED-BCIS2 was from 40 sites exclusively in the UK, whereas participants in STICHES were enrolled from 127 sites in 26 countries. Details of the propensity score-matched populations are reported in Supplementary data online, *Tables S4*–*S6*.

**Table 1:** Baseline characteristics of the pooled population

	CABG + MT STICHES *n* = 610	PCI + MT REVIVED *n* = 347	MT STICHES *n* = 602	MT REVIVED *n* = 353	Total *n* = 1912	*P*-value
Age, years	60.6 ± 9.0	70.0 ± 9.0	60.0 ± 9.6	68.8 ± 9.1	63.6 ± 10.2	<.001
Male sex, *n*(%)	537 (88)	302 (87)	527 (88)	312 (88)	1678 (88)	.962
Ethnicity, *n* (%)						<.001
White	407 (67)	306 (88)	420 (70)	328 (93)	1461 (76)	
Asian	107 (18)	32 (9)	102 (17)	17 (5)	258 (14)	
Black	18 (3)	3 (1)	13 (2)	3 (1)	37 (2)	
Mixed/other	78 (13)	6 (2)	67 (11)	5 (1)	156 (8)	
Body mass index, kg/m^2^	27.2 ± 4.7	28.4 ± 5.5	27.4 ± 4.9	28.7 ± 5.4	27.8 ± 5.1	<.001
Heart rate, b.p.m.	76 ± 16	70 ± 12	74 ± 14	69 ± 12	73 ± §4	<.001
Systolic blood pressure, mmHg	121 ± 18	125 ± 20	121 ± 17	125 ± 20	123 ± 19	<.001
Diastolic blood pressure, mmHg	75 ± 11	71 ± 12	76 ± 11	72 ± 12	74 ± 14	<.001
Current smoking, *n* (%)	130 (21)	61 (18)	122 (20)	75 (21)	388 (20)	.470
Diabetes, *n* (%)	240 (39)	136 (39)	238 (40)	153 (43)	767 (40)	.427
Chronic kidney disease, *n* (%)	49 (8)	73 (21)	45 (8)	56 (16)	223 (12)	<.001
Previous MI, *n* (%)	462 (76)	175 (50)	472 (78)	197 (56)	1306 (68)	<.001
Previous PCI, *n* (%)	82 (13)	66 (19)	74 (12)	76 (22)	298 (16)	<.001
Previous CABG, *n* (%)	22 (4)	12 (4)	14 (2)	22 (6)	70 (4)	.034
Peripheral vascular disease, *n* (%)	89 (15)	48 (14)	95 (16)	46 (13)	278 (15)	.295
No. of diseased coronary arteries, *n* (%)						*<*.*001*
0/1	148 (24)	36 (10)	159 (26)	39 (11)	382 (20)	
2	233 (38)	178 (51)	229 (38)	166 (47)	806 (42)	
3	229 (38)	133 (38)	214 (36)	148 (42)	724 (38)	
LVEF, %	26.7 (6.1)	27.0 (6.6)	26.4 (6.0)	27.0 (6.9)	26.7 (6.3)	.438
LVESVi, mL/m^−2^	84.5 (42)	69.1 (30)	83.3 (41)	68.3 (28)	77.5 (37)	<.001
NYHA class, *n* (%)						<.001
I/II	384 (63)	265 (77)	381 (63)	248 (70)	1278 (67)	
III/IV	226 (37)	80 (23)	221 (37)	102 (29)	629 (33)	
CCS class, *n* (%)						<.001
0	217 (36)	228 (66)	225 (37)	236 (67)	906 (48)	
I/II	361 (59)	111 (32)	351 (58)	107 (31)	930 (49)	
III/IV	32 (5)	7 (2)	26 (4)	8 (2)	73 (4)	
RAAS inhibitor, *n* (%)*	567 (93)	308 (89)	544 (90)	317 (90)	1736 (91)	.840
Beta-blocker, *n* (%)	507 (83)	315 (91)	529 (88)	319 (90)	1670 (87)	.001
Mineralocorticoid receptor antagonist, *n* (%)	280 (46)	176 (51)	276 (46)	170 (48)	902 (47)	.13
Implantable cardioverter–defibrillator, *n* (%)	15 (3)	77 (22)	14 (2)	71 (20)	177 (9)	<.001

*P*-values report between-trial comparisons.

CABG + MT STICHES, coronary artery bypass grafting plus medical therapy in STICHES; CCS, Canadian Cardiovascular Society; LVEF, left ventricular ejection fraction; LVESVi, left ventricular end-systolic volume index; MI, myocardial infarction; MT REVIVED, medical therapy in REVIVED-BCIS2; MT STICHES, medical therapy in STICHES; NYHA, New York Heart Association; RAAS, renin–angiotensin–aldosterone system; PCI + MT REVIVED, percutaneous coronary intervention plus medical therapy in REVIVED-BCIS2.

The median (inter-quartile range) duration of follow-up was 118 (109–132) months in STICHES and 41 (28–60) months in REVIVED-BCIS2. Follow-up for the primary outcome was ascertained at final follow-up in 1881 patients (98.3%). In total, 1117 participants (58.4%) experienced a primary outcome event at a mean of 57 ± 41 months; unadjusted event rates were 404 (66%) in CABG + MT STICHES, 129 (37%) in PCI + MT REVIVED, 450 (75%) in MT STICHES, and 134 (38%) in MT REVIVED. Unadjusted event rates for secondary outcomes are summarized in Supplementary data online, *Table S7*.

### Primary outcome

**Figure 1:**
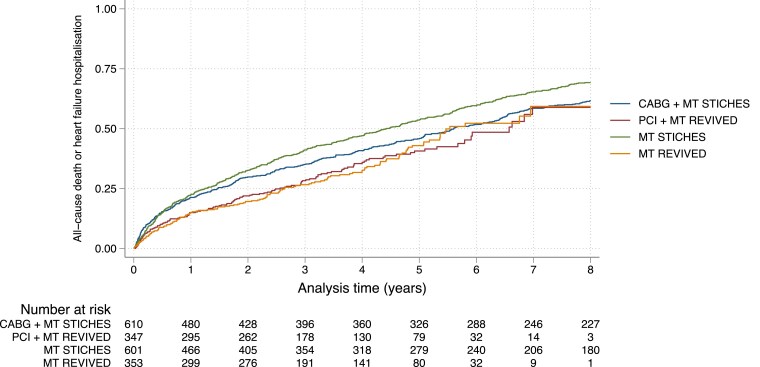
Kaplan–Meier plot of event-free survival for the primary outcome of all-cause death or hospitalization for heart failure in the whole population. CABG + MT STICHES, coronary artery bypass grafting plus medical therapy in STICHES; MT REVIVED, medical therapy in REVIVED-BCIS2; MT STICHES, medical therapy in STICHES; PCI + MT REVIVED, percutaneous coronary intervention plus medical therapy in REVIVED-BCIS2

**Figure 2:**
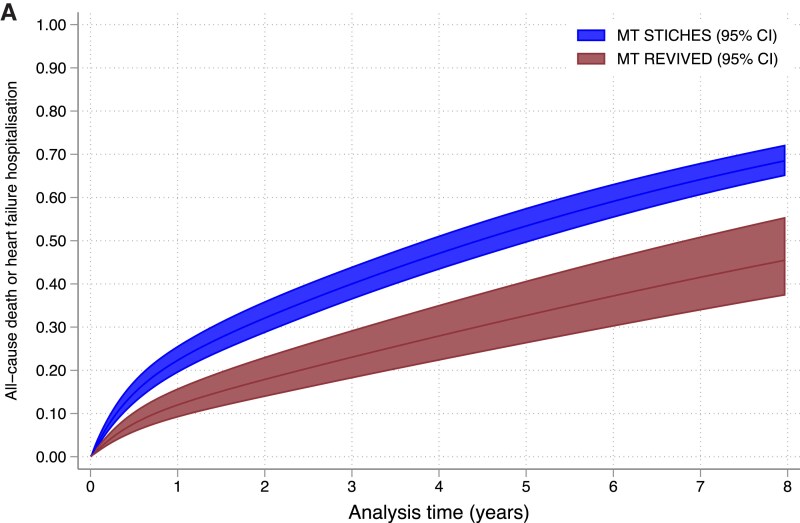

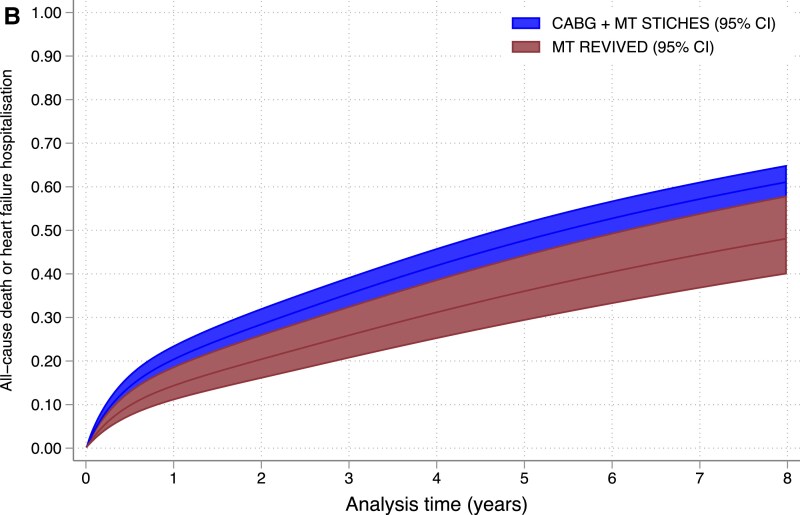

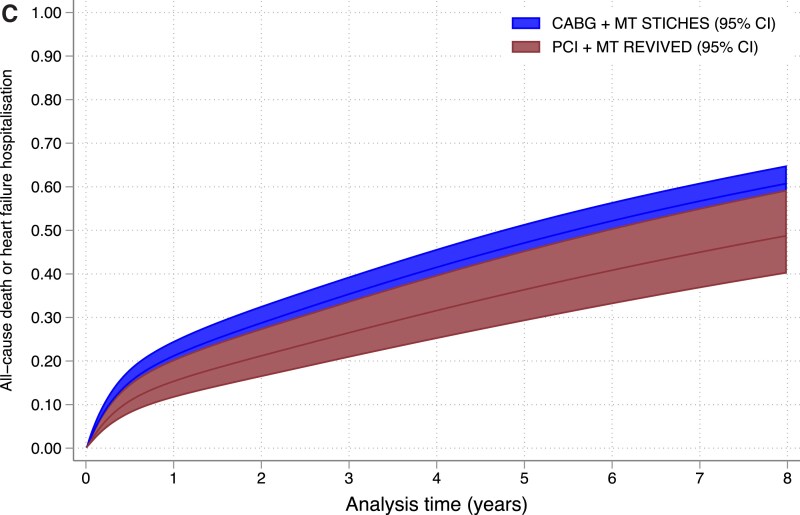
Parametric survival analysis comparing occurrence of the primary outcome between (*A*) MT REVIVED and MT STICHES, (*B*) MT REVIVED and CABG + MT STICHES, and (*C*) PCI + MT REVIVED and CABG + MT STICHES in the 1-to-*N* propensity score-matched population. CABG + MT STICHES, coronary artery bypass grafting plus medical therapy in STICHES; CI, confidence interval; MT REVIVED, medical therapy in REVIVED-BCIS2; MT STICHES, medical therapy in STICHES; PCI + MT REVIVED, percutaneous coronary intervention plus medical therapy in REVIVED-BCIS2

In the adjusted analysis of the whole study population, participants in PCI + MT REVIVED and MT REVIVED were less likely to experience a primary outcome event than those in MT STICHES (HR 0.59, 95% CI 0.47–0.74, *P* < .001 and HR 0.60, 95% CI 0.48–0.74, *P* < .001, respectively, *Figure 1* and *Table 2*). The propensity score-matched analysis confirmed these results, with participants in MT REVIVED less likely to experience a primary outcome event than participants in MT STICHES (HR 0.46, 95% CI 0.34–0.62, *P* < .001, *Figure 2* and *Table 2*). The predicted median event-free survival time was 145 (95% CI 95–196) months in MT REVIVED and 57 (95% CI 47–64) months in MT STICHES, a difference of 88 months.

**Table 2:** The primary outcome of all-cause death or hospitalization for heart failure in the pooled and propensity score-matched populations

	Hazard ratio	95% CI	*P*-value
Pooled, adjusted population			
MT STICHES	Reference group		
CABG + MT STICHES	0.80	0.70–0.92	.001
PCI + MT REVIVED	0.59	0.47–0.74	<.001
MT REVIVED	0.60	0.48–0.74	<.001
Propensity score-matched population			
MT REVIVED vs. MT STICHES	0.46	0.34–0.62	<.001
MT REVIVED vs. CABG + MT STICHES	0.62	0.45–0.84	.002
PCI + MT REVIVED vs. CABG + MT STICHES	0.61	0.43–0.87	.007

CABG + MT STICHES, coronary artery bypass grafting plus medical therapy in STICHES; CI, confidence interval; MT REVIVED, medical therapy in REVIVED-BCIS2, MT STICHES, medical therapy in STICHES; PCI + MT REVIVED, percutaneous coronary intervention plus medical therapy in REVIVED-BCIS2.

Participants in CABG + MT STICHES experienced a greater number of primary outcome events than those in MT REVIVED (*Figure 1* and *Table 2*). In those who received revascularization, enrolment in CABG + MT STICHES was not associated with incremental benefit, compared with PCI + MT REVIVED (*Figure 1* and *Table 2*). The results were consistent in the propensity score-matched population (*Figure 2*).

### Secondary outcome

The number of deaths from any cause, cardiovascular deaths, first hospitalizations for heart failure, and myocardial infarctions were 982 (51.4%), 708 (37.0%), 463 (24.2%), and 147 (7.7%), respectively. The occurrence of all-cause and cardiovascular death was higher in the MT STICHES alone compared with CABG + MT STICHES, PCI + MT REVIVED, or MT REVIVED alone (with similar rates in the latter groups) (*Table 3*). Compared with CABG + MT STICHES, first hospitalization for heart failure was more frequently observed in MT STICHES but less frequently observed in both PCI + MT REVIVED and MT REVIVED (*Table 3*). Assignment to CABG + MT STICHES was associated with a lower hazard for myocardial infarction compared with assignment to MT STICHES, PCI + MT REVIVED, or MT REVIVED (*Table 3*).

**Table 3:** Secondary outcomes in the pooled and propensity score-matched populations

Secondary outcomes	Hazard ratio	95% CI	*P*-value
**All-cause death**			
Pooled, adjusted population			
MT STICHES	Reference group		
CABG + MT STICHES	0.83	0.72–0.97	.015
PCI + MT REVIVED	0.67	0.52–0.85	.001
MT REVIVED	0.71	0.56–0.90	.004
Propensity score-matched population			
MT REVIVED vs. MT STICHES	0.60	0.42–0.84	.003
MT REVIVED vs. CABG + MT STICHES	0.68	0.49–0.95	.023
PCI + MT REVIVED vs. CABG + MT STICHES	0.67	0.46–0.96	.031
**Cardiovascular death**			
Pooled, adjusted population			
MT STICHES	Reference group		
CABG + MT STICHES	0.78	0.66–0.92	.004
PCI + MT REVIVED	0.67	0.50–0.89	.007
MT REVIVED	0.78	0.59–1.02	.070
Propensity score-matched population			
MT REVIVED vs. MT STICHES	0.73	0.49–1.07	.108
MT REVIVED vs. CABG + MT STICHES	0.87	0.58–1.30	.497
PCI + MT REVIVED vs. CABG + MT STICHES	0.79	0.51–1.22	.286
**Hospitalization for heart failure**			
Pooled, adjusted population			
MT STICHES	Reference group		
CABG + MT STICHES	0.71	0.57–0.87	.001
PCI + MT REVIVED	0.47	0.33–0.67	<.001
MT REVIVED	0.47	0.33–0.66	<.001
Propensity score-matched population			
MT REVIVED vs. MT STICHES	0.38	0.24–0.59	<.001
MT REVIVED vs. CABG + MT STICHES	0.60	0.37–0.97	.037
PCI + MT REVIVED vs. CABG + MT STICHES	0.40	0.23–0.70	.001
**Myocardial infarction**			
Pooled, adjusted population			
MT STICHES	Reference group		
CABG + MT STICHES	0.61	0.37–0.98	.042
PCI + MT REVIVED	1.87	1.09–3.19	.023
MT REVIVED	1.92	1.14–3.24	.015
Propensity score-matched population			
MT REVIVED vs. MT STICHES	2.30	1.13–4.67	.021
MT REVIVED vs. CABG + MT STICHES	3.86	1.58–9.40	.003
PCI + MT REVIVED vs. CABG + MT STICHES	3.65	1.27–10.46	.016

CABG + MT STICHES, coronary artery bypass grafting plus medical therapy in STICHES; CI, confidence interval; MT REVIVED, medical therapy in REVIVED-BCIS2; MT STICHES, medical therapy in STICHES; PCI + MT REVIVED, percutaneous coronary intervention plus medical therapy in REVIVED-BCIS2.

In the propensity score-matched analysis for secondary outcomes, participants in PCI + MT REVIVED were less likely to die from any cause than in the CABG + MT STICHES group (*Table 3*), with similar trends for MT REVIVED vs. MT STICHES and MT REVIVED vs. CABG + MT STICHES. No between-group differences were observed for cardiovascular death (*Table 3*). Hospitalization for heart failure occurred less often in REVIVED-BCIS2 patients than in either arm of STICHES. Enrolment in CABG + MT STICHES was associated with a lower risk of myocardial infarction than both REVIVED-BCIS2 groups across all comparisons.

### Sensitivity analysis

Baseline characteristics of STICHES patients recruited in North America, Western Europe, and Poland are reported in Supplementary data online, *Table S8*. In the sensitivity analysis incorporating only these patients, both the PCI + MT REVIVED and MT REVIVED groups had lower rates of the primary outcome than patients in MT STICHES (HR 0.57, 95% CI 0.44–0.73, *P* < .001 and HR 0.58, 95% CI 0.45–0.74, *P* < .001, respectively, Supplementary data online, *Figure S5* and *Table S9*). The occurrence of the primary outcome did not differ between CABG + MT STICHES and MT STICHES in this subpopulation (HR 0.88, 95% CI 0.73–1.05, *P* = .162). The results were consistent in the propensity score-matched populations (see Supplementary data online, *Table S9*).

## Discussion

Our key findings are that in patients with ischaemic LV dysfunction (i) outcomes of patients treated with MT alone were better in REVIVED-BCIS2 than in STICHES, (ii) a strategy of CABG and MT in STICHES was not associated with an incremental survival benefit when compared with MT alone in REVIVED-BCIS2, and (iii) a strategy of CABG and MT in STICHES was not associated with an incremental benefit in all-cause mortality and heart failure hospitalization, when compared with PCI and MT as delivered in REVIVED-BCIS2 (*Structured Graphical Abstract*).

This is the first analysis to utilize individual participant data from randomized trials that investigated the role of coronary revascularization in ischaemic LV dysfunction. Notwithstanding the fact that allocation to revascularization or medical therapy was by randomization, whereas between-trial comparisons are observational, this highly curated and independently adjudicated dataset affords unique insights into the prognostic impact of evolution of medical therapy as well as the relative efficacy of the revascularization strategies. The close reproduction of the initial within-trial results in the propensity matched population supports our statistical approach and the robustness of this analysis.

Our key finding of marked improvement in outcomes of medically treated patients is striking though consistent with the wider heart failure literature.^11,12^ In the two decades between the recruitment of the first STICHES participant and final follow-up of REVIVED-BCIS2, development of new pharmacological treatment continued at pace. This included the first randomized trial evidence for eplerenone, sacubitril/valsartan, and the sodium–glucose cotransporter 2 inhibitors dapagliflozin and empagliflozin.^13–16^ Whilst it might be expected that outcomes of the control groups in these pharmacologic trials also improved over time, ours is the first observation of this effect specific to patients with ischaemic LV dysfunction considered for revascularization. Utilization of implantable cardiac device therapy also increased substantially over this period, as evidenced by the fact that approximately half of all patients in REVIVED-BCIS2 have received an implantable cardioverter–defibrillator by the end of follow-up. Our estimate of 88 additional months of event-free survival between the two trials is particularly notable given the characteristics of patients enrolled in REVIVED-BCIS2. Participants were an average of 10 years older with a higher prevalence of chronic kidney disease, both of which are key predictors of all-cause mortality.

Whilst we have hypothesized that the improvement in outcomes is due to an improvement in medical therapy, the observed differences may relate to other unmeasured differences in the trial populations. For instance, REVIVED-BCIS2 recruited solely in the UK, whilst STICHES was an international trial recruiting in 26 countries with potentially wider variation in health systems and social determinants of care. These differences may have affected event rates and treatment effects, given that development in systems of care, the widespread deployment of multidisciplinary teams, and community heart failure services have been shown to reduce mortality and rehospitalizsation.^17^ Although we adjusted for measured baseline confounders, unmeasured baseline differences in the populations may also underlie the observed differences, including differences in the trial inclusion criteria. REVIVED-BCIS2 required the presence of extensive myocardial viability for enrolment, and whilst secondary analyses of STICHES indicate that most patients in STICHES had extensive viability, it is possible that the inclusion of those without viability creates a higher-risk population.^18,19^ Likewise, the need for mitral valve surgery was an exclusion from REVIVED-BCIS2 but not from STICHES, and the representation of patients with significant valve disease could also lead to worse observed outcomes.

Another postulated explanation for the different results of the REVIVED and STICHES trials was that PCI and CABG are mechanistically distinct treatment modalities and hence might be associated with different long-term prognostic impact. Although there have been numerous randomized trials comparing PCI with CABG, reduced LVEF has been a ubiquitous exclusion criterion.^20–22^ Our finding that PCI in REVIVED was associated with better outcomes than CABG in STICHES should be interpreted with the important caveat that we have compared strategies rather than revascularization modalities *per se*; adjunctive medical therapy is an integral component of these strategies in both trials. Given the margin of prognostic difference in the medical therapy used in the two trials, the comparisons between CABG and PCI arms are speculative and should be treated as hypothesis generating only. Furthermore, the previously referenced trials comparing CABG and PCI have generally favoured CABG, as have meta-analyses and observational studies in populations with reduced LVEF.^23,24^ A recent *in silico* analysis leveraging routinely collected data from more than 13 000 patients in England who matched the STICHES inclusion criteria concluded that PCI was associated with a significantly higher rate of mortality and cardiovascular hospitalization than CABG.^25^ A meta-analysis of five randomized trials of coronary revascularization in chronic heart failure due to CAD showed a lower risk of all-cause mortality, but that the difference was neither substantial nor robust; the authors concluded that further trials were required.^26^

Ultimately, two key questions remain. The first is whether CABG remains superior to contemporary medical therapy alone in patients with ischaemic LV dysfunction and stable CAD of the type recruited to REVIVED-BCIS2 and STICH. The MASS-VI (HF) trial (ISRCTN77449548), a single centre randomized trial assessing the incremental value of CABG in 600 patients with ischaemic LV dysfunction with evidence of ischaemia myocardial viability who are receiving medical therapy, has recently been completed. The primary outcome is a composite of all-cause death, non-fatal myocardial infarction, stroke, and unstable angina requiring reintervention. The results are anticipated in September 2025.^27^ How definitively this trial changes the evidence base will depend on the characteristics of the enrolled population and generalizability of results from a single centre to international practice.

The second question is a robust comparison of the two revascularization modalities in a head-to-head trial that seeks to answer the question of whether CABG is superior to PCI in patients with CAD, LV dysfunction, and a clear indication for revascularization (predominantly angina and acute coronary syndromes) who are receiving optimal medical therapy. A family of harmonized randomized trials is currently underway (ISRCTN29654606, NCT05427370,^28^ and NCT05584280) or planned, under the umbrella of the STICH3 consortium. These trials will be unique as the first direct comparison of CABG and PCI in this patient population, reflecting a randomized evolution of the current data, albeit with a subtle difference in indication.

Our study has some limitations. As discussed above, the between-trial comparisons are observational and we can only hypothesize as to the drivers of difference in outcome. Given the striking difference in outcome between medical therapy groups, this is particularly relevant to observations involving CABG and PCI which should only be considered hypothesis generating; we can neither make statements on the incremental benefit of CABG over contemporary medical therapy, nor the relative benefits of PCI and CABG in this context. Second, due to limitations in the trial CRF data collection, we were not able to fully quantify changes in medical therapy: whilst the prescribed medication classes were recorded, details on doses were not collected in either trial. Newer agents such as angiotensin receptor–neprilysin inhibitors and sodium–glucose cotransporter 2 inhibitors were not available during STICHES or the early phase of REVIVED-BCIS2; their unmeasured use later in REVIVED-BCIS2 may account for some of the difference in outcomes of medically treated patients. Data on statins and antiplatelets were also not included in the combined trial eCRF, though there is limited evidence of an outcome benefit of these agents in a heart failure population. There were significant differences in the period of enrolment, health systems, and age of the populations. Although we attempted to control for measured confounding, we were constrained by available information, and there is always unmeasured confounding risk in observational studies that use propensity matching approaches. Our pre-specified plan for adjustment and propensity matching did not include blood pressure or creatinine, which are powerful markers of prognosis and may have improved the analysis, though the populations were generally well matched. We analysed randomized clinical trials which collected identical or very similar information, although we acknowledge that all changes in practice over time will not have been necessarily captured in the datasets. For the survival models that included all four arms, we could not account for random effects (intercept) across the two trials. Our initial analysis plan was to use the first 5 years of STICH trial data; however, we revised the analysis plan early on to include the extended follow-up data, to make maximal use of the available data. The resulting difference in follow-up time may have influenced the findings, though our time-to-event analysis should minimize the potential impact of this difference, particularly given the treatment effect of CABG in STICH was consistent over time, with a statistically significant benefit developing due to the larger number of events with longer follow-up. There was a strong predominance of white and male patients in both trials, and applicability to female patients and other ethnicities is less certain. There were some differences in outcome definitions and adjudication which may have impacted the secondary outcomes. The change in biomarkers used to diagnose myocardial infarction from STICHES (creatine kinase myocardial band (CK-MB) and low-sensitivity troponin assays) to REVIVED-BCIS2 (high-sensitivity troponin assays) may have affected the frequency of this outcome. Finally, patients with a recent acute coronary syndrome or limiting angina were excluded (per-protocol criteria and/or as applied by investigators) from both STICHES and REVIVED-BCIS2 and hence the results of our analysis should not be extrapolated to these clinical scenarios.

## Conclusions

Medical therapy in the recent REVIVED trial was associated with better outcomes than medical therapy in the older STICHES trial, in keeping with the use of more prognostically impactful therapies over time. The relative efficacy of revascularization modalities needs to be compared directly in patients with ischaemic LV dysfunction, and in these trials, all patients should receive contemporary medical therapy. Likewise, consideration should be given to updating the evidence for CABG against contemporary medical therapy in this population.

## Supplementary Material

ehaf080_Supplementary_Data

## Declarations

### Data Availability

The data supporting this study will be made available on reasonable request to the corresponding author, 1 year after publication.

### Pre-registered Clinical Trial Number

The pre-registered clinical trial numbers are NCT 01920048 (REVIVED-BCIS2) and NCT 00023595 (STICHES).
