# Supplementary material for: Medical therapy and outcomes in REVIVED-BCIS2 and STICHES: an individual patient data analysis
Source: Eur Heart J. 2025 Mar 6;46(22):2052–62. doi: 10.1093/eurheartj/ehaf080 (PMC12146480; doi:10.1093/eurheartj/ehaf080)
Supplement: ehaf080_Supplementary_Data [file ehaf080_supplementary_data.docx]

**Supplementary Appendix**

**Table S1 – Inclusion and exclusion criteria**

| **REVIVED-BCIS2** | **STICH** |
| --- | --- |
| Inclusion | |
| - Left ventricular ejection fraction ≤ 35% - Extensive coronary artery disease (British Cardiovascular Intervention Society Jeopardy Score ≥ 6) - Viability in at least four dysfunctional myocardial segments amenable to treatment with PCI | - Left ventricular ejection fraction ≤ 35% - Coronary artery disease amenable to CABG - Absence of left main coronary artery disease (stenosis < 50%) - Absence of CCS III angina or greater (markedly limiting ordinary activity) |
| Exclusion | |
| - Myocardial infarction < 4 weeks previously - Decompensated heart failure requiring inotropic support, invasive or non-invasive ventilation or intra-aortic balloon pump/left ventricular assist device therapy < 72 hours prior to randomisation - Valve disease requiring intervention - Contraindications to PCI - Previously enrolled in REVIVED-BCIS2 or current enrolment in another study that may affect REVIVED-BCIS2 outcome data - Life expectancy < 1 year due to non-cardiac pathology - Age < 18 years - Sustained ventricular tachycardia/fibrillation or appropriate implantable cardioverter defibrillator discharge < 72 hours prior to randomisation - eGFR <25 ml/min, unless established on dialysis - Women who are pregnant | - Recent acute myocardial infarction judged to be an important cause of left ventricular dysfunction - Cardiogenic shock (within 72 hours of randomization), as defined by the need for intra-aortic balloon support or requirement for intravenous inotropic support - Aortic valvular heart disease clearly indicating need for aortic valve repair or replacement - Plan for percutaneous intervention of coronary artery disease - Current participation in another clinical trial in which a patient is taking an investigational drug or receiving an investigational medical device - Noncardiac illness with life expectancy of less than 3 years - Failure to provide informed consent - History of more than 1 prior coronary artery bypass operation - Noncardiac illness imposing substantial operative morbidity - Conditions/circumstances likely to lead to poor treatment adherence - Previous heart, kidney, liver or lung transplantation |

**Table S2 – Outcome definitions**

| **Outcome** | **REVIVED** | **STICH** |
| --- | --- | --- |
| Cardiovascular death | All deaths where there is no clinical or post-mortem evidence of a non-cardiovascular aetiology | Death will be considered to be of cardiac etiology unless there is an obvious non-cardiac cause. Defined as sudden death, or death attributed to recurrent myocardial infarction, heart failure, a cardiovascular procedure, stroke, or other cardiovascular etiology |
| Heart failure hospitalization | Hospital admission (lasting >24 hours) for deteriorating symptoms or signs of heart failure, where there is a documented diagnosis of heart failure and the patient receives initiation or intensification of treatment for heart failure. Initiation or intensification of treatment includes at least one of the following:   - increase in oral diuretic dose or addition of another oral diuretic; - intravenous vasoactive therapy (vasodilator, inotrope or vasopressor); - mechanical circulatory support (MCS) (including intra-aortic balloon pump (IABP), Impella, extra-corporeal membrane oxygenation (ECMO)); or - cardiac transplantation.   Elective admission for implantation or revision of ICD/cardiac resynchronisation therapy (CRT) devices will NOT constitute an endpoint. | Congestive Heart Failure (CHF) will be adjudicated in the case of an unplanned presentation for new or worsening heart failure requiring an overnight stay in which the patient receives treatment with parenteral therapy including diuretics, inotropic, or vasodilator agents. In the absence of documentation of these therapies, description of significant diuresis will be considered as criteria for heart failure therapy. |
| Acute myocardial infarction | The MI should be given one of the following five classifications:  **1. Spontaneous MI (>48 hours after PCI/CABG)**  Detection of a rise and/or fall of cardiac biomarkers (preferably Trop-T or I), with at least one value higher than the 99th percentile upper reference limit (URL)*) AND symptoms consistent with ischaemia OR dynamic ECG changes (including >1mm ST elevation, new Left Bundle Branch Block (LBBB) >1mm ST depression, >3mm T wave inversion).  **2. Peri-procedural MI (<48 hours after PCI/CABG)**  *Following PCI:* Trop-T or I >5 times the 99th percentile URL (or 5 times the baseline value if this is higher than the URL) in combination with any of the following: (i) evidence of prolonged ischaemia (>20 min) as demonstrated by prolonged chest pain and/or ischaemic ST changes; (ii) new pathological Q waves; (c) angiographic evidence of a flow limiting complication, such as of loss of patency of a side branch, persistent slow-flow or no-reflow, embolisation; or (d) imaging evidence of new loss of viable myocardium or new regional wall motion abnormality.  *Following CABG:* Trop-T or I >10 times the 99th percentile URL (or 10 times the baseline value if this is higher than the URL) in combination with any of the following: (i) new pathological Q waves; (ii) angiographically documented new graft or new native coronary artery occlusion; or (iii) imaging evidence of new loss of viable myocardium or new regional wall motion abnormality.  **3. Sudden death**  Cardiac arrest accompanied by new ST elevation/LBBB on ECG and/or evidence of fresh coronary thrombus at autopsy/angiography.  **4. Probable MI**  Features suggestive of MI but evidence not available.  **5. No MI**  No evidence of MI. | To be adjudicated as a STICH hospitalization for myocardial infarction, a patient must have an increase in cardiac enzymes and at least one of the two following criteria:  • Typical clinical presentation Or  • Typical ECG changes (evolving ST-segment or T-wave changes in two or more contiguous leads, the development of Q waves in two or more contiguous leads, or the development of new left branch bundle block).  Increase in cardiac enzymes as defined as any one of the following:  • CK – MB greater than twice the upper limit of normal (ULN)  • TNT or I greater than three times the ULN.  An MI that occurs after a coronary bypass procedure or SVR will be adjudicated as a STICH myocardial infarction only if there are new Q-waves present and the CK-MB is greater than 5 times the ULN and two times the pre – surgery level for CABG. |

**Table S3 – Missingness of Data**

|  | **Complete** | **Incomplete** | **Imputed** |
| --- | --- | --- | --- |
| **STICHES (n = 1212)** | | |  |
| BMI | 1212 | 0 | 0 |
| SBP | 1212 | 0 | 0 |
| DBP | 1212 | 0 | 0 |
| HR | 1212 | 0 | 0 |
| LVEF | 1212 | 0 | 0 |
| Smoking | 1211 | 1 | 1 |
| Hypertension | 1212 | 0 | 0 |
| CKD | 1211 | 1 | 1 |
| ACE | 1212 | 0 | 0 |
| Vessels | 1211 | 1 | 1 |
| NYHA | 1212 | 0 | 0 |
| CCS | 1212 | 0 | 0 |
| **REVIVED (n = 700)** | | |  |
| BMI | 696 | 4 | 4 |
| SBP | 698 | 2 | 2 |
| DBP | 698 | 2 | 2 |
| HR | 698 | 2 | 2 |
| LVEF | 698 | 2 | 2 |
| Smoking | 700 | 0 | 0 |
| Hypertension | 699 | 1 | 1 |
| CKD | 691 | 9 | 9 |
| ACE | 697 | 3 | 3 |
| Vessels | 700 | 0 | 0 |
| NYHA | 695 | 5 | 5 |
| CCS | 697 | 3 | 3 |

Table S3. Counts of missing data in the STICH and REVIVED-BCIS2 trial datasets. ACE – angiotensin converting enzyme inhibitor, BMI – body mass index, CCS – Candian Cardiovascular Society angina score, CKD -chronic kidney disease, DBP – diastolic blood pressure, HR- heart rate, LVEF – left ventricular ejection fraction, NYHA – new York heart association, SBP – systolic blood pressure.

**Table S4 - Propensity score matching for MT STICHES vs. MT REVIVED**

|  | **MT STICHES**  **(N = 540)** | **MT REVIVED**  **(N = 201)** | **SMD** |
| --- | --- | --- | --- |
| **Characteristics** | | | |
| Age (SD) | 61.1 (9.3) | 65.2 (8.8) | -0.41 |
| Male sex – N (%) | 471 (87) | 179 (89) | -0.07 |
| Diabetes – N (%) | 217 (40) | 83 (41) | 0.08 |
| Chronic kidney disease – N (%) | 44 (8) | 23 (11) | 0.14 |
| Left ventricular ejection fraction - % (SD) | 26 (6) | 27 (7) | -0.10 |
| **Unadjusted events** | | | |
| All-cause death or hospitalization for heart failure – N (%) | 411 (76) | 74 (37) |  |
| All-cause death – N (%) | 365 (68) | 62 (31) |  |
| Cardiovascular death – N (%) | 266 (49) | 29 (24) |  |
| Heart failure hospitalization – N (%) | 188 (35) | 31 (15) |  |
| Myocardial infarction – N (%) | 41 (8) | 20 (10) |  |

Table S4. Unadjusted characteristics and events in the propensity score matched population. Note that median follow up times differed between trials. MT – medical therapy, N – number, R – REVIVED-BCIS2, SD – standard deviation, SMD – standardised mean difference, S – STICH.

**Table S5 – Propensity score matching for CABG + MT STICHES vs. MT REVIVED**

|  | **CABG + MT STICHES (N = 563)** | **MT REVIVED (N = 199)** | **SMD** |
| --- | --- | --- | --- |
| **Characteristics** | | | |
| Age (SD) | 61.2 (9) | 65.4 (9) | 0.02 |
| Male sex – N (%) | 498 (89) | 175 (88) | -0.02 |
| Diabetes – N (%) | 229 (41) | 84 (42) | 0.00 |
| Chronic kidney disease – N (%) | 47 (8) | 24 (12) | 0.03 |
| Left ventricular ejection fraction (%) | 27 (6) | 26 (7) | -0.06 |
| **Unadjusted events** | | | |
| All-cause death or hospitalization for heart failure – N (%) | 373 (66) | 73 (37) |  |
| All-cause death – N (%) | 333 (59) | 60 (30) |  |
| Cardiovascular death – N (%) | 227 (40) | 50 (25) |  |
| Heart failure hospitalization – N (%) | 146 (26) | 32 (16) |  |
| Myocardial infarction – N (%) | 27 (5) | 19 (10) |  |

Table S5. Unadjusted characteristics and events in the propensity score matched population. Note that median follow up times differed between trials. MT – medical therapy, N – number, R – REVIVED-BCIS2, SD – standard deviation, SMD – standardised mean difference, S – STICH.

**Table S6 – Propensity score matching for CABG + MT STICH vs. PCI + MT REVIVED**

|  | **CABG + MT STICHES (N = 543)** | **PCI + MT REVIVED (N = 184)** | **SMD** |
| --- | --- | --- | --- |
| **Characteristics** | | | |
| Age (SD) | 61.7 (9) | 65.6 (9) | -0.02 |
| Male sex – N (%) | 477 (88) | 168 (91) | -0.19 |
| Diabetes – N (%) | 218 (40) | 76 (47) | 0.05 |
| Chronic kidney disease – N (%) | 49 (9) | 29 (16) | 0.11 |
| Left ventricular ejection fraction (%) | 27 (6) | 27 (7) | -0.16 |
| **Unadjusted events** | | | |
| All-cause death or hospitalization for heart failure – N (%) | 370 (68) | 58 (32)) |  |
| All-cause death – N (%) | 335 (62) | 49 (27) |  |
| Cardiovascular death – N (%) | 235 (43) | 39 (21) |  |
| Heart failure hospitalization – N (%) | 141 (26) | 24 (13) |  |
| Myocardial infarction – N (%) | 27 (5) | 17 (9) |  |

Table S6. Unadjusted characteristics and events in the propensity score matched population. Note that median follow up times differed between trials. MT – medical therapy, N – number, R – REVIVED-BCIS2, SD – standard deviation, SMD – standardised mean difference, S – STICH.

**Table S7 – Primary and secondary outcomes in the pooled population**

|  | **STICH-CABG**  **N = 610** | **REVIVED-PCI**  **N = 347** | **STICH-MT**  **N = 602** | **REVIVED-MT**  **N = 353** | **Total**  **N = 1912** |
| --- | --- | --- | --- | --- | --- |
| **Primary outcome** | | | | | |
| All-cause death or hospitalisation for heart failure – N (%) | 404 (66) | 129 (37) | 450 (75) | 134 (38) | 1117 (58) |
| **Secondary outcomes** | | | | | |
| All-cause death – N (%) | 359 (59) | 110 (32) | 398 (66) | 115 (33) | 982 (51) |
| Cardiovascular death – N (%) | 247 (41) | 76 (22) | 297 (49) | 88 (25) | 708 (37) |
| Hospitalisation for heart failure – N (%) | 157 (26) | 51 (15) | 201 (33) | 54 (15) | 463 (24) |
| Myocardial infarction – N (%) | 30 (5) | 37 (11) | 42 (7) | 38 (11) | 147 (8) |

Table S7. Unadjusted primary and secondary outcome events in the pooled population. Note that median follow up times differed between trials. MT – medical therapy, N – number.

**Table S8 – Characteristics of STICHES patients enrolled in North America, Western Europe and Poland**

|  | CABG+MT STICHES  N = 328 | MT STICHES  N = 321 |
| --- | --- | --- |
| Age – yr | 62 (9) | 61 (10) |
| Male sex – n(%) | 287 (88) | 280 (87) |
| Ethnicity – n (%)  White  Asian  Black  Mixed/other | 297 (91)  3 (1)  14 (4)  14 (4) | 303 (94)  0 (0)  9 (3)  9 (3) |
| Body mass index – kg.m^-2^ | 27.7 (4.8) | 28.3 (5.1) |
| Heart rate - bpm | 75 (18) | 73 (14) |
| Systolic blood pressure - mmHg | 120 (18) | 119 (17) |
| Diastolic blood pressure - mmHg | 74 (11) | 73 (11) |
| Current smoking – n (%) | 66 (20) | 65 (20) |
| Diabetes – n (%) | 139 (42) | 131 (41) |
| Chronic kidney disease – n (%) | 34 (10) | 29 (9) |
| Previous MI – n (%) | 251 (77) | 256 (80) |
| Previous PCI – n (%) | 57 (17) | 54 (17) |
| Previous CABG – n (%) | 10 (3) | 7 (2) |
| Peripheral vascular disease – n (%) | 53 (16) | 48 (15) |
| Number of diseased coronary arteries – n (%)  0/1  2  3 | 78 (24)  120 (37)  130 (40) | 88 (27)  111 (35)  122 (38) |
| LVEF - % | 26.5 (6.2) | 26.1 (6.1) |
| NYHA class – n (%)  I/II  III/IV | 218 (66)  110 (34) | 210 (65)  111 (35) |
| CCS class – n (%)  0  I/II  III/IV | 137 (42)  170 (52)  21 (6) | 133 (41)  174 (54)  14 (4) |
| RAAS inhibitor – n (%)* | 309 (94) | 301 (94) |
| Beta blocker – n (%) | 284 (87) | 296 (92) |
| Implantable cardioverter defibrillator – n (%) | 11 (3) | 13 (4) |

Table 1. Baseline characteristics of the pooled population. P-values report between trial comparisons. BPM – beats per minute, CABG+MT STICHES – coronary artery bypass grafting plus medical therapy in STICHES, CCS – Canadian cardiovascular society, LVEF – left ventricular ejection fraction, LVESVi – left ventricular end systolic volume index, MI – myocardial infarction, MT STICHES – medical therapy in STICHES, NYHA – New York Heart Association, RAAS – renin-angiotensin-aldosterone system.

**Table S9 – Primary outcomes in STICHES patients enrolled in North America, Western Europe and Poland and in REVIVED patients**

| **Primary outcome** | | | |
| --- | --- | --- | --- |
| **All-cause death or hospitalization for heart failure** | | | |
|  | Hazard ratio | 95% CI | p-value |
| Pooled, adjusted population | | | |
| MT STICHES | Reference group | | |
| CABG+MT STICHES | 0.88 | 0.73 to 1.05 | 0.162 |
| PCI+MT REVIVED | 0.57 | 0.44 to 0.73 | < 0.001 |
| MT REVIVED | 0.58 | 0.45 to 0.74 | < 0.001 |
| Propensity score-matched population | | | |
| MT REVIVED vs. MT STICHES | 0.61 | 0.42 to 0.88 | 0.009 |
| MT REVIVED vs. CABG+MT STICHES | 0.61 | 0.42 to 0.89 | 0.009 |
| PCI+MT REVIVED vs. CABG+MT STICHES | 0.47 | 0.32 to 0.69 | < 0.001 |

CABG+MT STICHES – coronary artery bypass grafting plus medical therapy in STICHES, CI – confidence interval, MT REVIVED – medical therapy in REVIVED-BCIS2, MT STICHES – medical therapy in STICHES, PCI+MT REVIVED – percutaneous coronary intervention plus medical therapy in REVIVED-BCIS2

**Figure S1 – Secondary outcomes**

**
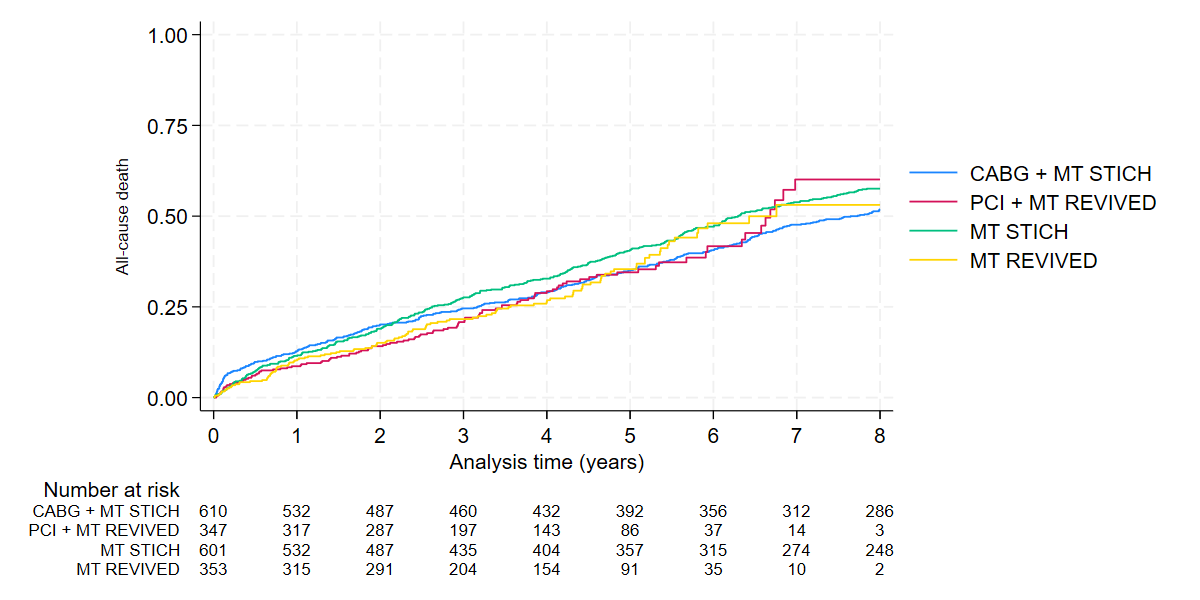
**

**
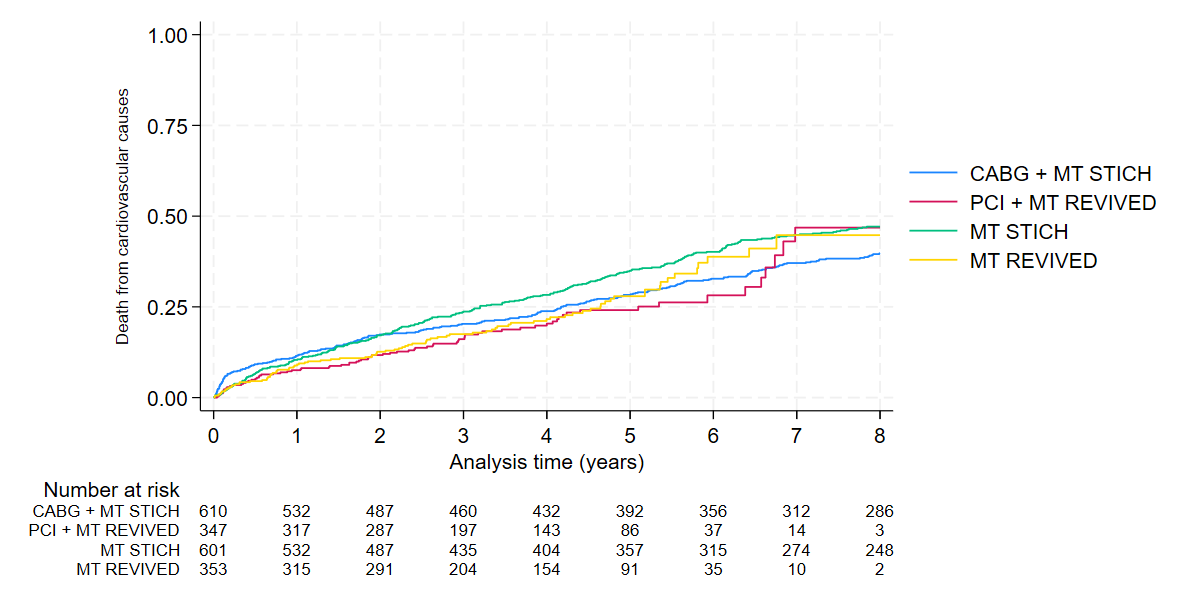
**

**
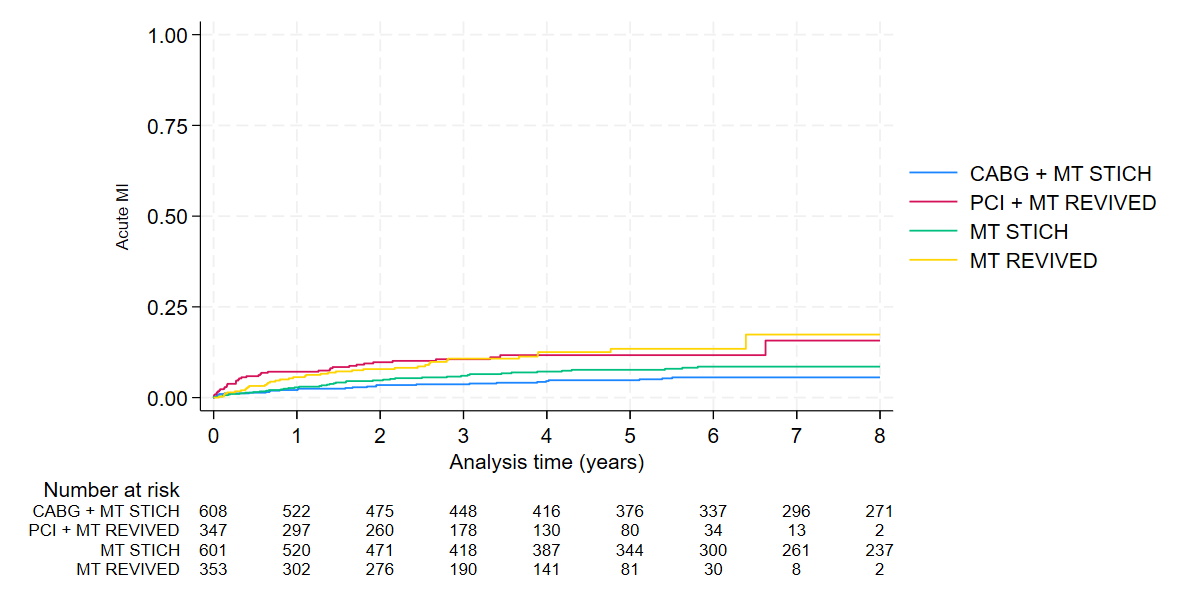
**

Figure 1 - Kaplan Meier plots of event-free survival for the secondary outcomes of all-cause death, cardiovascular death, hospitalization for heart failure and acute myocardial infarction in the whole population. CABG + MT STICH – coronary artery bypass grafting plus medical therapy in STICHES , MT REVIVED – medical therapy in REVIVED-BCIS2, MT STICH – medical therapy in STICHES, PCI + MT REVIVED – percutaneous coronary intervention plus medical therapy in REVIVED-BCIS2.

**Figure S1 – Propensity matched population: all-cause death**

**
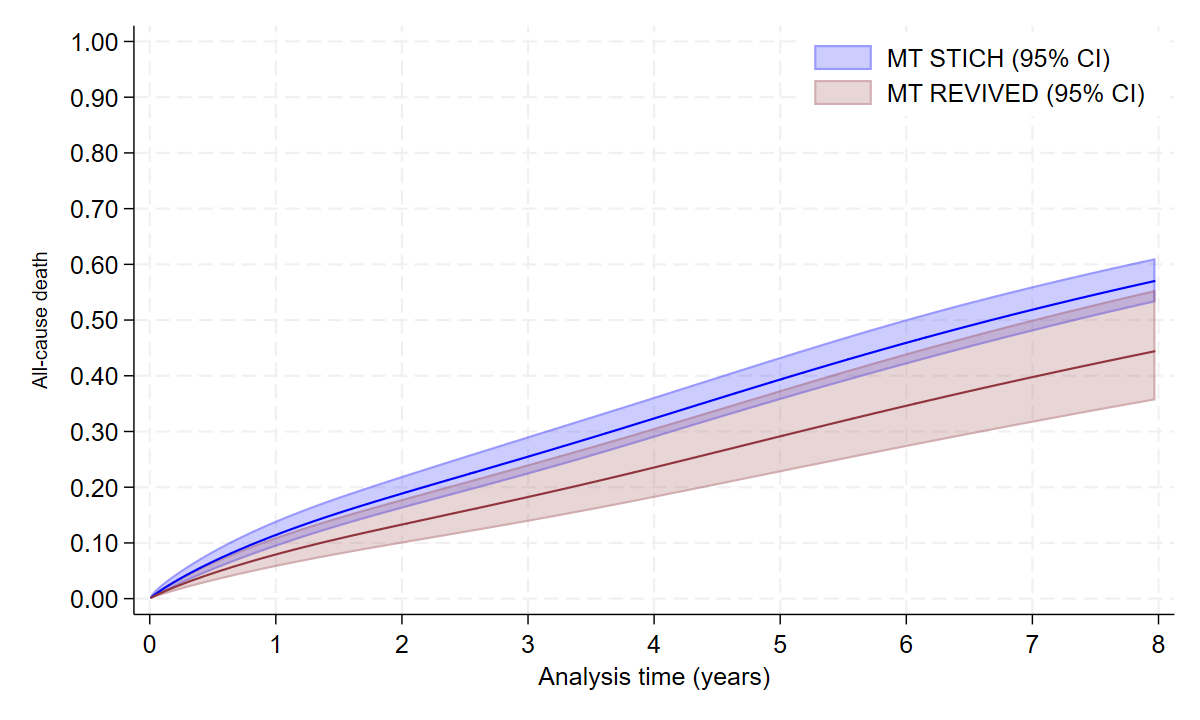
**

**
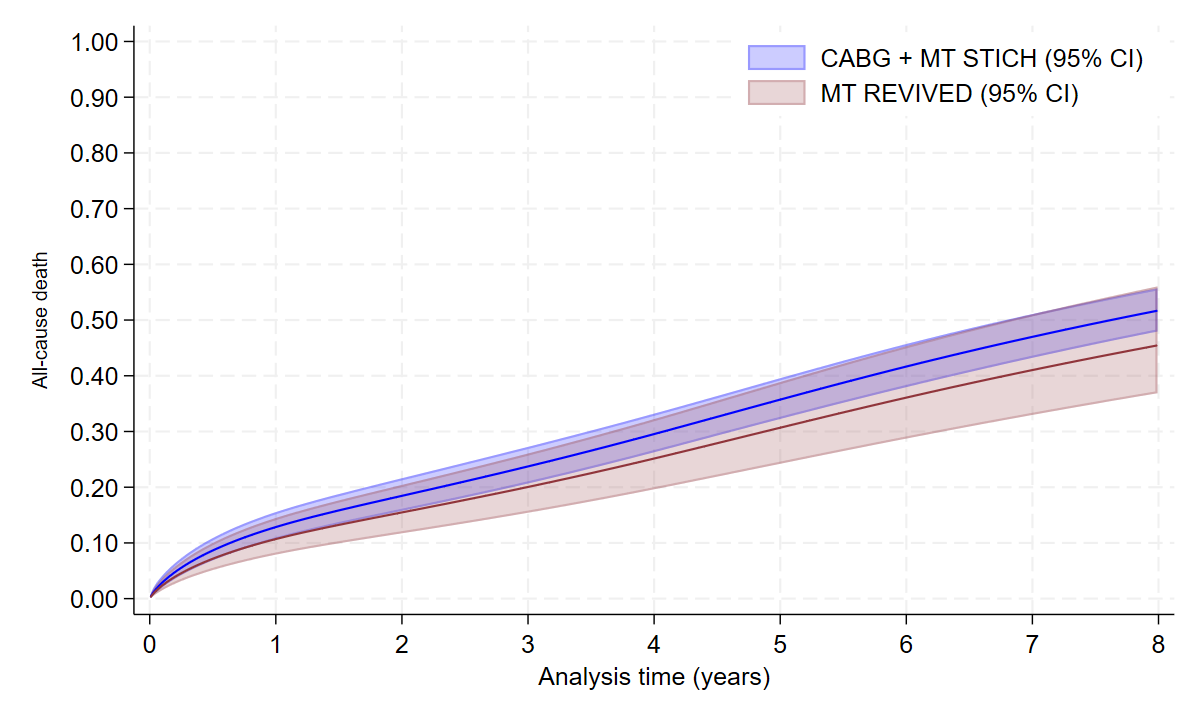
**

**
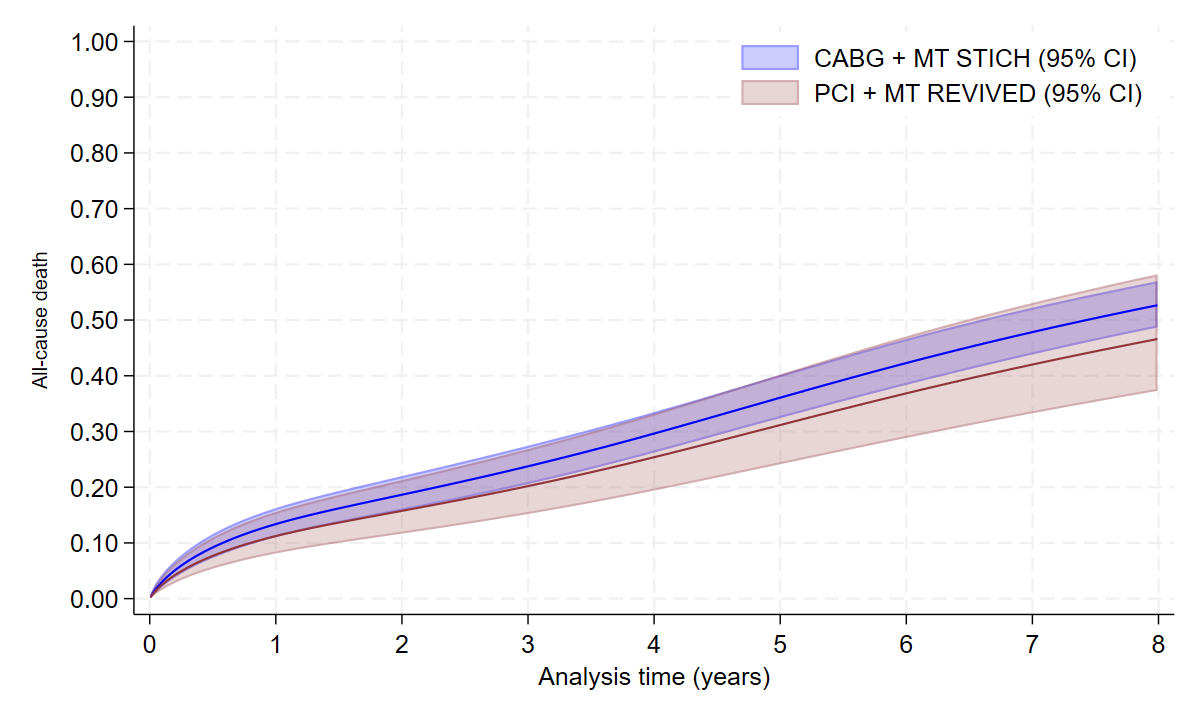
**

Figure S1 - Parametric survival analysis comparing occurrence of all-cause death between top) MT REVIVED and MT STICH, middle) PCI + MT REVIVED and CABG + MT STICH, and bottom) MT REVIVED and CABG + MT STICH in the 1-to-N propensity score matched population. CABG – coronary artery bypass grafting, CI – confidence interval, MT – medical therapy, PCI – percutaneous coronary intervention, R – REVIVED-BCIS2 trial, S – STICH trial.

**Figure S2 – Propensity matched population: cardiovascular death**

**
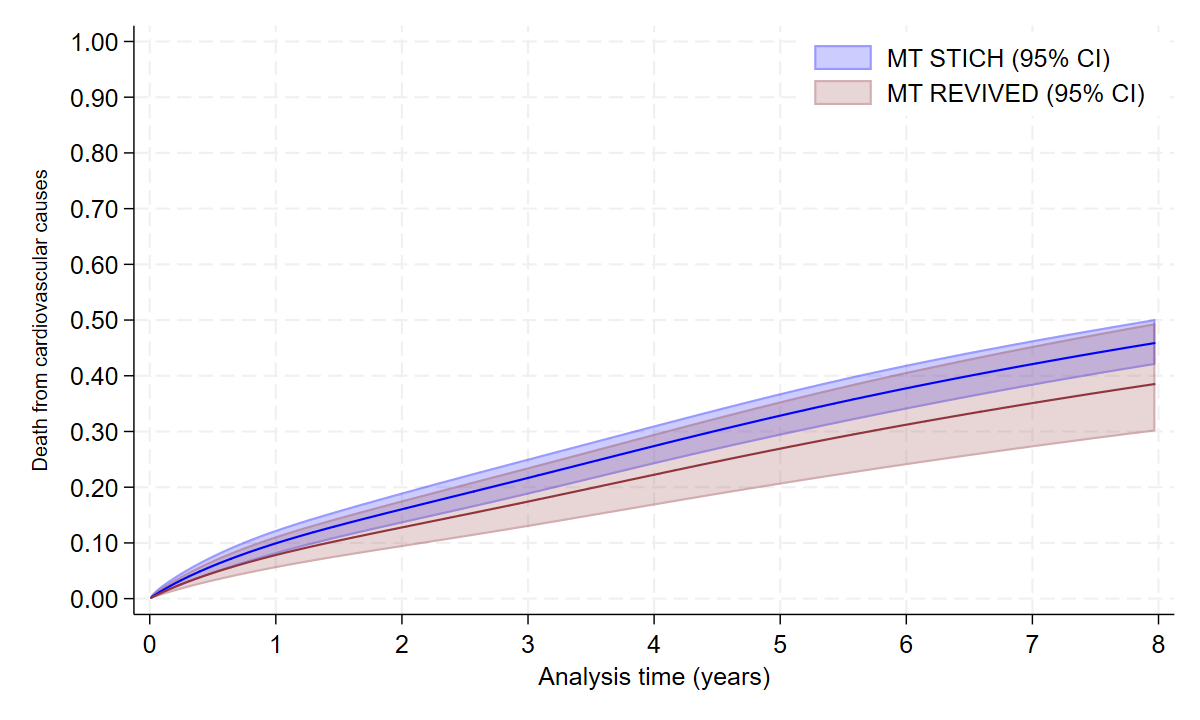
**

**
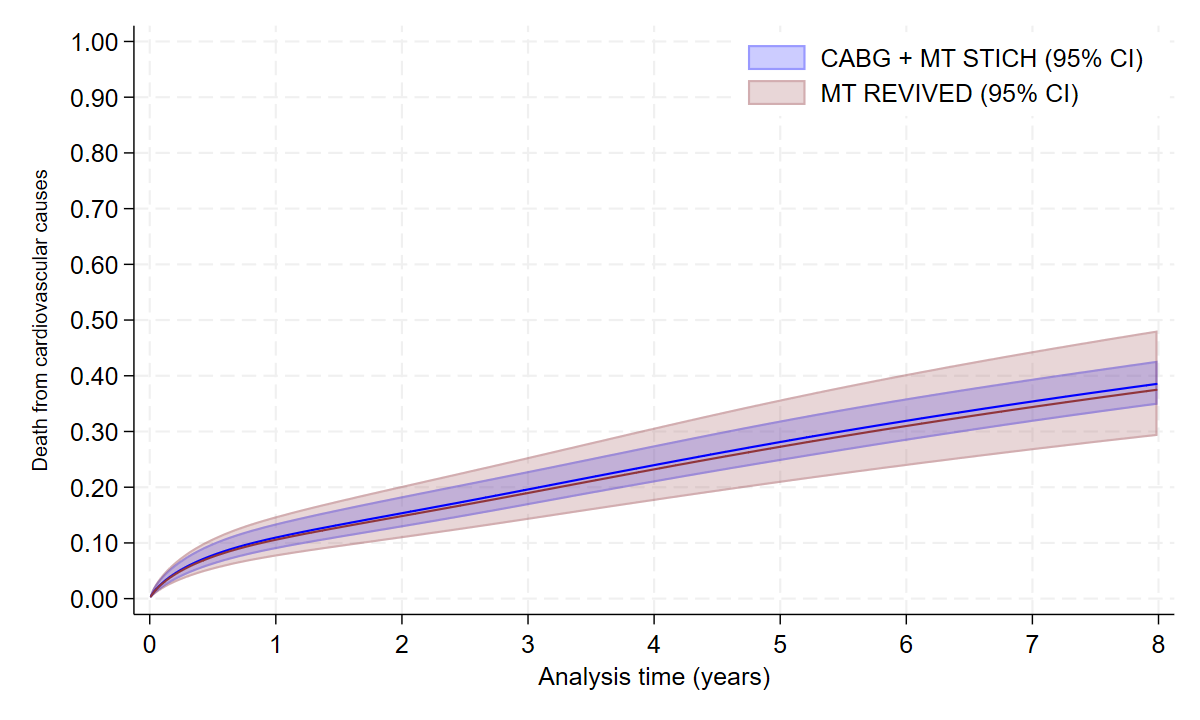
**

**
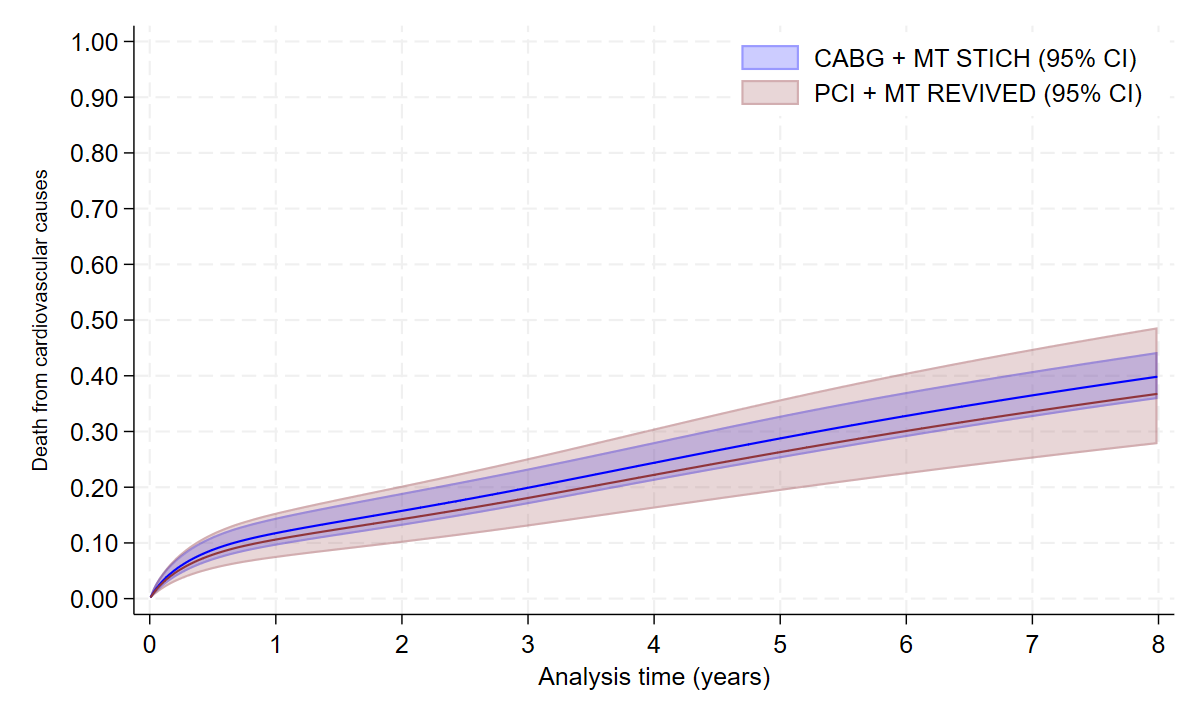
**

Figure S2 - Parametric survival analysis comparing occurrence of cardiovascular death between top) MT REVIVED and MT STICH, middle) PCI + MT REVIVED and CABG + MT STICH, and bottom) MT REVIVED and CABG + MT STICH in the 1-to-N propensity score matched population. CABG – coronary artery bypass grafting, CI – confidence interval, MT – medical therapy, PCI – percutaneous coronary intervention, R – REVIVED-BCIS2 trial, S – STICH trial.

**Figure S3 – Propensity matched population: hospitalization for heart failure**


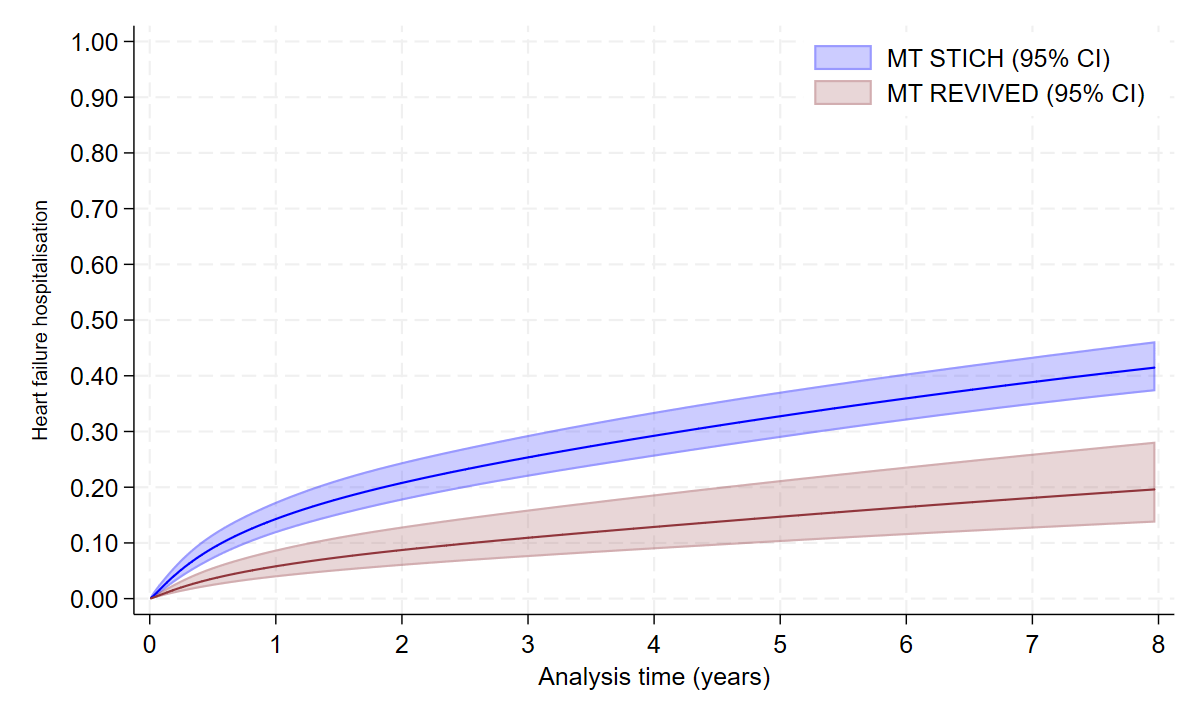


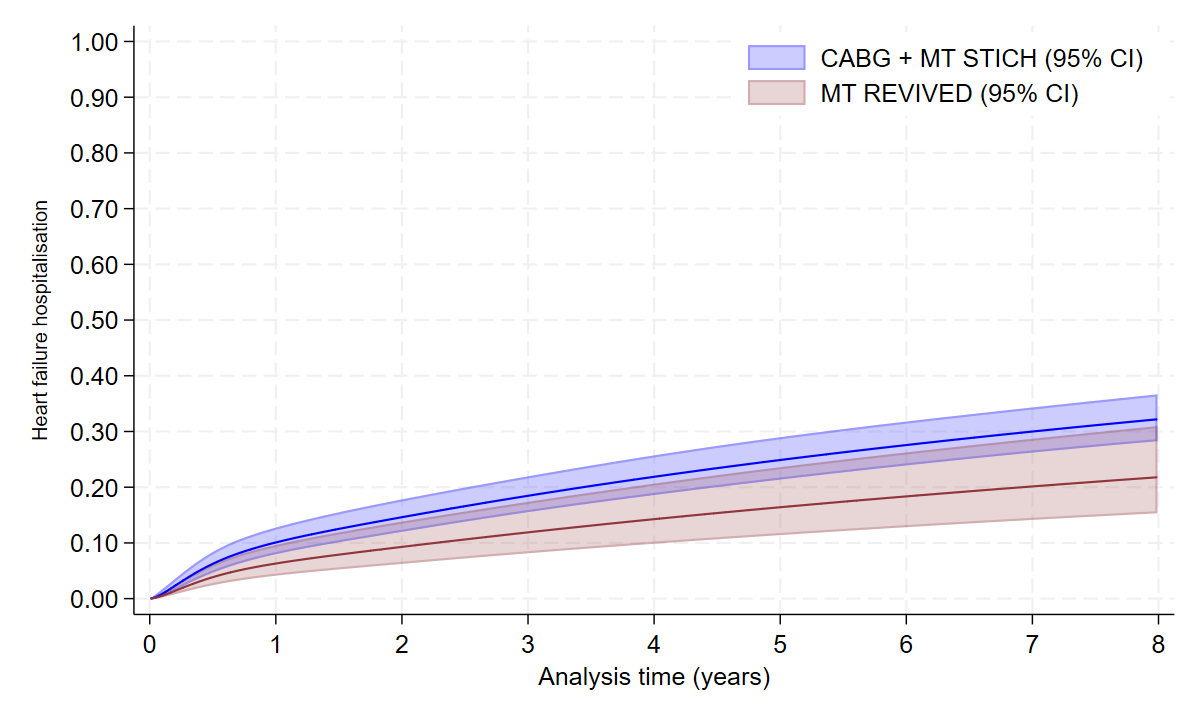


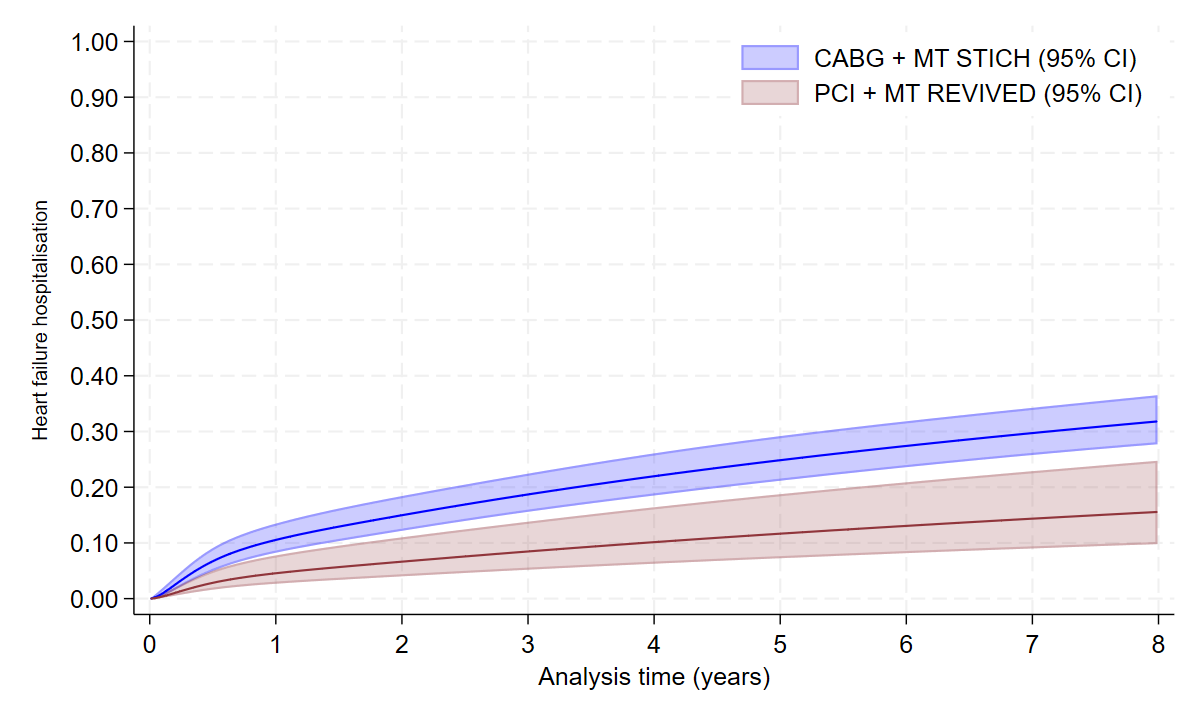


Figure S3 - Parametric survival analysis comparing occurrence of hospitalisation for heart failure between top) MT REVIVED and MT STICH, middle) PCI + MT REVIVED and CABG + MT STICH, and bottom) MT REVIVED and CABG + MT STICH in the 1-to-N propensity score matched population. CABG – coronary artery bypass grafting, CI – confidence interval, MT – medical therapy, PCI – percutaneous coronary intervention, R – REVIVED-BCIS2 trial, S – STICH trial.

**Figure S4 – Propensity matched population: myocardial infarction**


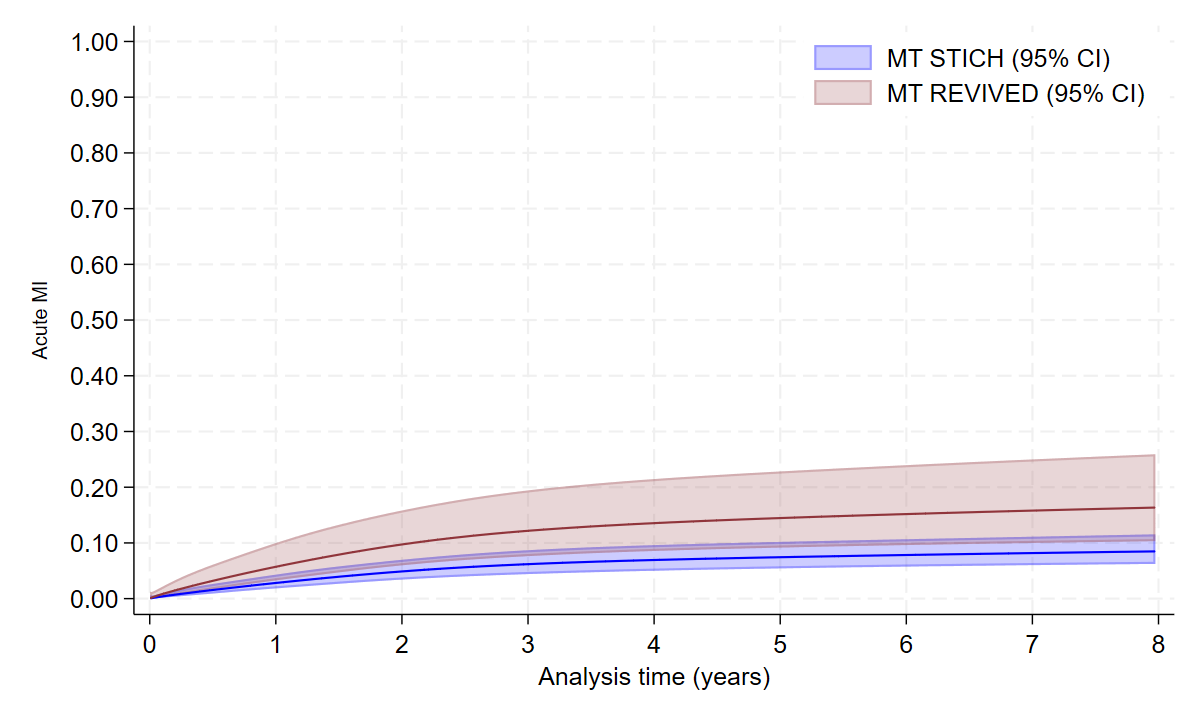


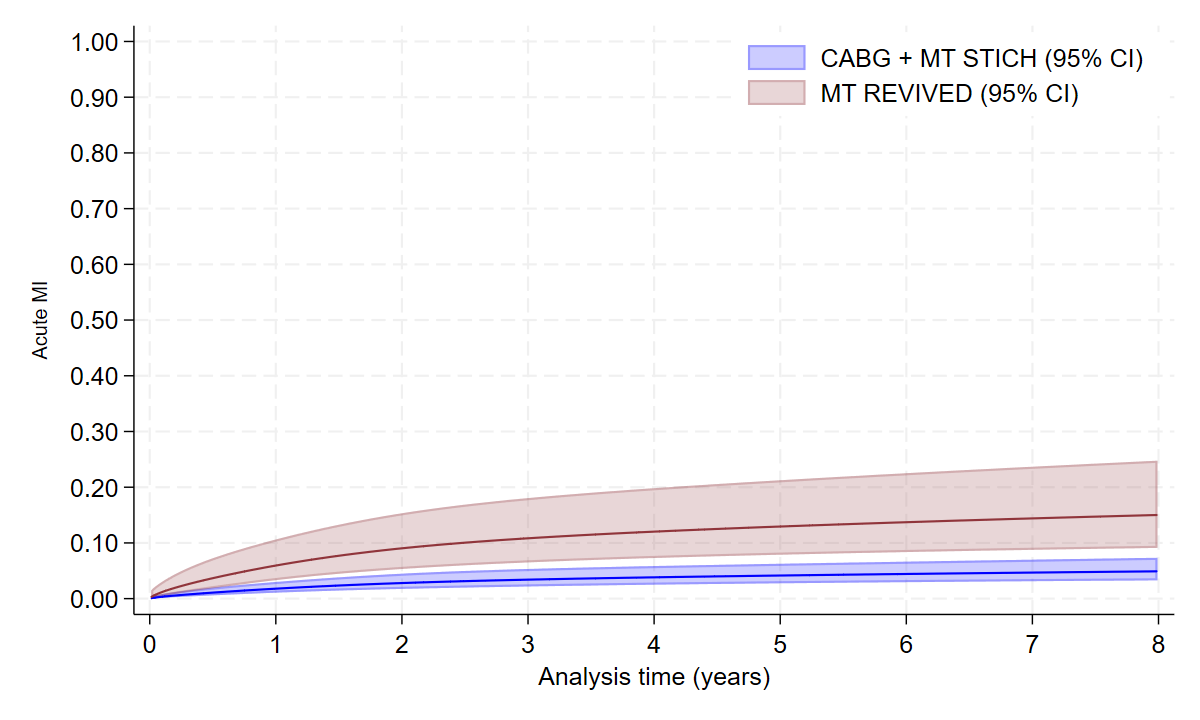

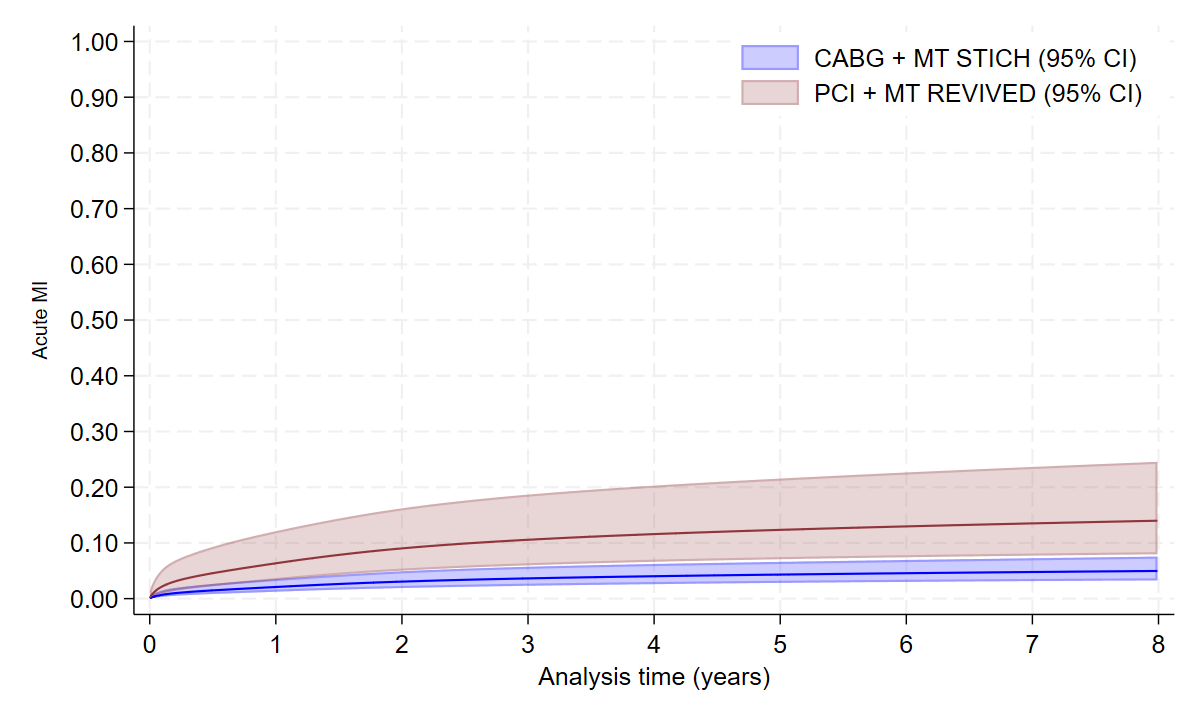


Figure S4 - Parametric survival analysis comparing occurrence of myocardial infarction between top) MT REVIVED and MT STICH, middle) PCI + MT REVIVED and CABG + MT STICH, and bottom) MT REVIVED and CABG + MT STICH in the 1-to-N propensity score matched population. CABG – coronary artery bypass grafting, CI – confidence interval, MT – medical therapy, PCI – percutaneous coronary intervention, R – REVIVED-BCIS2 trial, S – STICH trial.

**Figure S5**


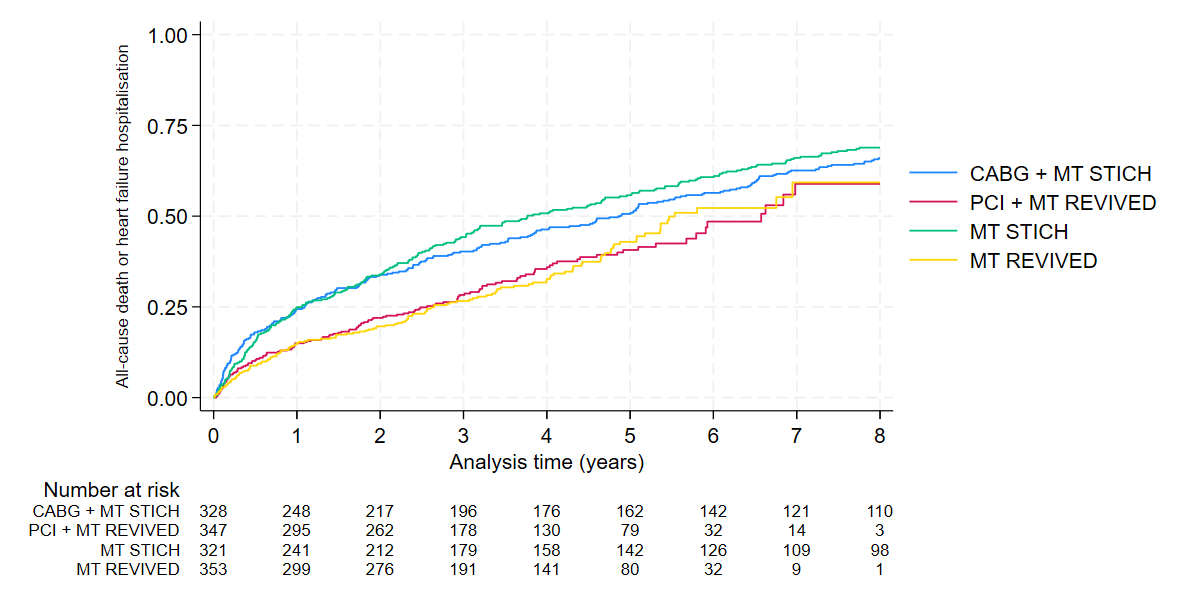


Figure S5 - Kaplan Meier plot of event-free survival for the primary outcome of all-cause death or hospitalization for heart failure, with the STICHES population restricted to North America, Western Europe and Poland. CABG+MT STICHES – coronary artery bypass grafting plus medical therapy in STICHES , MT REVIVED – medical therapy in REVIVED-BCIS2, MT STICHES – medical therapy in STICHES, PCI+MT REVIVED – percutaneous coronary intervention plus medical therapy in REVIVED-BCIS2.
